# Identifying epilepsy based on machine‐learning technique with diffusion kurtosis tensor

**DOI:** 10.1111/cns.13773

**Published:** 2021-12-23

**Authors:** Li Kang, Jin Chen, Jianjun Huang, Tijiang Zhang, Jiahui Xu

**Affiliations:** ^1^ College of Electronics and Information Engineering Shenzhen University Shenzhen China; ^2^ The Guangdong Key Laboratory of Intelligent Information Processing Shenzhen China; ^3^ The Affiliate Hospital of Zunyi Medical University Zunyi China

**Keywords:** DKI, kurtosis tensor, machine learning, MRI negative

## Abstract

**Introduction:**

Epilepsy is a serious hazard to human health. Minimally invasive surgery is an extremely effective treatment to refractory epilepsy currently if the location of epileptic foci is given. However, it is challenging to locate the epileptic foci since a multitude of patients are MRI‐negative. It is well known that DKI (diffusion kurtosis imaging) can analyze the pathological changes of local tissues and other regions of epileptic foci at the molecular level. In this article, we propose a new localization way for epileptic foci based on machine‐learning method with kurtosis tensor in DKI.

**Methods:**

We recruited 59 children with hippocampus epilepsy and 70 age‐ and sex‐matched normal controls; their T1‐weighted images and DKI were collected simultaneously. Then, the hippocampus in DKI is segmented based on a mask as a local brain region, and DKE is utilized to estimate the kurtosis tensor of each subject's hippocampus. Finally, the kurtosis tensor is fed into SVM (support vector machine) to identify epilepsy.

**Results:**

The classifier produced 95.24% accuracy for patient versus normal controls, which is higher than that obtained with FA (fractional anisotropy) and MK (mean kurtosis). Experimental results show that the kurtosis tensor is a kind of remarkable feature to identify epilepsy, which indicates that DKI images can act as an important biomarker for epilepsy from the view of clinical diagnosis.

**Conclusion:**

Although the classification task for epileptic patients and normal controls discussed in this article did not directly achieve the location of epileptic foci and only identified epilepsy on certain brain region, the epileptic foci can be located with the results of identifying results on other brain regions.

## Graphical abstract

A machine‐learning method is proposed to recognize the epileptic patients. The hippocampus of DKI for each patient and normal control is extracted with generated mask, and then, its kurtosis tensor is obtained with DKE, which is subsequently fed into the SVM to identify epilepsy. The classifier can produce 95.24% accuracy for patient versus normal controls, which is higher than that obtained with FA (fractional anisotropy) and MK (mean kurtosis). These results demonstrate that the kurtosis tensor of DKI is more effective than the parameters derived from the DKI.

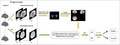

## 1 INTRODUCTION

Epilepsy is one of the most common diseases of the nervous system in the elderly, second to dementia and stroke,^1^ and is characterized by recurrent, unpredictable spontaneous epileptic seizures. Epidemiological data indicate that about 70 million people worldwide suffer from epilepsy.^2^ In China, the prevalence of active epilepsy is 0.48%–8.5%, and about 9 million people on the mainland suffer from epilepsy. Long‐term recurrent seizures can lead to progressive brain tissue damage and cognitive impairment in patients with epilepsy,^3^, ^4^ which are highly dangerous. Temporal lobe epilepsy is one of the most common focal epilepsy, and the most common pathological change is hippocampal sclerosis.^5^, ^6^ For MRI‐positive temporal lobe epilepsy patients, because of clinical characteristics of unilateral temporal lobe hypometabolism, it is easy to diagnose and treatment. However, it is challenging to locate the lesion since some patients with temporal lobe epilepsy are MRI‐negative, which cannot be captured by conventional MRI. Conventional MRI‐negative epilepsy accounts for 30% of the epilepsy population and up to 80% of the first seizure epilepsy patients.^7^

The etiology of epilepsy is extremely complex and includes abnormal neurotransmitter signaling, reactive glial cell proliferation, and altered synaptic structure. Minimally invasive surgery is currently an effective treatment for drug‐refractory epilepsy, and preoperative lesion localization is the key to successful surgery. However, it is challenging to locate the epileptic foci since a multitude of patients are MRI‐negative. How to locate epileptic foci with available imaging techniques is a scientific problem of great practical value.^8^, ^9^

Researchers around the world carried out research aiming to find effective ways to locate epilepsy. Tan^10^ combined MRI and PET features to detect FCD patients by using SVM and image block‐based classifiers, and found that the detection results of both features were better than MRI and PET as features alone, with a sensitivity of 93%, higher than the latter two 82% and 68%. 3D arterial spin labeling is also employed by researchers to perform the localization of epilepsy,^11^ and they reported a 69.2% accuracy. At the same time, they found that 1H‐MRS (proton magnetic resonance spectroscopy) can locate epilepsy with 76.9% accuracy. Furthermore, when combining the two methods, 84.6% localization accuracy was achieved.

Actually, it remains arduous for the patients who are MRI‐negative to detect their lesion by conventional MRI since there are no obvious changes in the lesion. It is indispensable to detect the lesion with a kind of effective imaging technique. DTI (diffusion tensor imaging) and DKI (diffusion kurtosis imaging)^12^, ^13^, ^14^ are recently developed magnetic resonance imaging techniques, which use the anisotropy of water molecules in different tissues to reflect subtle structural and functional changes in tissues, and can detect early subtle lesions in brain tissue superior to structural images. Due to its ability to detect the subtle changes in brain tissue at the molecular level, DKI has shown their important scientific value in the study of the pathophysiological mechanisms of epilepsy and the lateralization and localization of epileptogenic foci,^15^, ^16^ and it has gradually increased in recent years in the diagnostic applications of epilepsy to accurately assess the presence of abnormalities in the gray and white matter of patients with epilepsy, quantify the microstructural abnormalities in the brain, and provide important information for the localization of epileptogenic foci.^17^, ^18^ It was found^19^ that patients with MRI‐negative temporal lobe epilepsy (MRI‐TLE) had microstructural white matter alterations, and showed significantly reduced FA values in the corpus callosum, bilateral superior and inferior fiber tracts, and the left corticospinal tract.

Although DKI has shown significant value in the localization and analysis of epileptogenic foci, however, due to the extremely large amount of data in functional MRI sequences, reliance on physician's review to analyze images cannot meet clinical requirements, which is not only time‐consuming and laborious but also prone to missed diagnoses and misdiagnoses.

To solve this problem, many scholars have tried to analyze medical images automatically with machine‐learning methods.^20^, ^21^, ^22^ Gaizo et al.^23^ used machine‐learning methods to classify epilepsy based on diffusion MRI, achieving accuracy of 68% (FA), 51% (MD, mean diffusion), and 82% (MK), and they also verified statistically that FA and MK are more significant than MD to diagnose epilepsy. In their study, the DKI images of the complete brain region were used to perform the classification task, but their work could only determine whether the epilepsy lesion is presented but cannot locate them. In contrast, Huang et al.^24^ proposed a method to locate epileptic foci for conventional MRI‐negative epileptic patients based on DKI and deep learning technique. In particular, a convolutional neural network (CNN) is introduced to segment the hippocampus, and VGG16 with transfer learning is used to characterize the image. The extraction of vectors uses feature vectors as the input of the SVM classification network, and the accuracy obtained by the method is much better than that of Gaizo et al., which improved by 19.16% (FA), 8.78% (MD), and 8.81% (MK).

In studies on localization of epileptogenic foci based on DKI, most of the literature utilized the parameters such as FA, MK and MD, which are derived from the kurtosis tensor of DKI to analyze the medical images.^25^, ^26^ The effectiveness of these parameters in locating epileptogenic foci has also been demonstrated in the published literature and in our experiments. However, it can be inferred that the kurtosis tensor itself contains more complete information than MK, MD, and FA parameters since the latter is derived from the former, and it is theoretically feasible to obtain higher accuracy for locating epileptogenic foci with kurtosis tensor. Based on this, this article proposes an automatic identification method with kurtosis tensor for epileptogenic foci localization based on machine‐learning technology, which is expected to improve the accuracy of localization of epileptogenic foci by using the kurtosis tensor as a comprehensive biomarker for classification under a machine‐learning framework.

## 2 MATERIALS AND METHODS

### 2.1 Subjects

The data used in this article, DKI and T1‐weighted images, were collected by the Affiliated Hospital of Zunyi Medical University, which include 59 patients (32 males and 27 females) with epilepsy lesions in the hippocampus; all patients were diagnosed according to the 2010 version diagnostic criteria of the ILAE by intermediate‐grade pediatricians or higher. All patients are conventional MRI‐negative, and in the next section, DKI in the hippocampal region will be studied by statistical methods to obtain the conclusion of abnormal microstructure. In addition, 70 healthy volunteers (37 males and 33 females) with matching gender, age, and education level were recruited as the normal control group. The subjects were all right‐handed, and we performed a statistical analysis of years onset, VIQ (Verbal Intelligence Quotient), PIQ (Performance Intelligence Quotient), and FIQ (Full‐scale Intelligence Quotient) in all patients with hippocampal epilepsy. The statistics of normal control and epilepsy patients by age are listed in Table 1.

**TABLE 1:** Statistical information of subjects

Item	Patients (*n* = 59)	Normal (*n* = 70)
Gender(M/F)	32/27	37/33
Age(years)	11.13 ± 2.89 (range 7–18)	12.82 ± 3.13 (range 7–18)
Handedness	59R	70R
Duration(years)	4.22 ± 3.15 (range 1–13)	—
VIQ	93.90 ± 18.99 (range 46–122)	—
PIQ	90.87 ± 18.99 (range 43–129)	—
FIQ	91.93 ± 19.36 (range 39–125)	—

### 2.2 Image acquisition

All participants underwent MRI examinations (3.0 T HDxt, GE Healthcare). Before starting the examination, remove the metal substance carried by the subject, wear earplugs to reduce noise, fix the subject's head to reduce head movement, and straighten the head. Participants were asked to close their eyes, lie down, and stay awake. The scanning range is the entire head.

Scanning parameters:

1. 3D T1BRAVO: repetition time = 7.8 ms, echo time = 3.0 ms, inversion time = 450 ms, flip angle = 15°, the field of view = 256 × 256 mm, spatial resolution: 1 × 1 × 1mm, slice thickness = 1 mm, slices = 256, scan time = 208 s.
2. DKI: *b* value (0, 1000, 2000 s/mm^2^), the diffusion‐sensitive gradient field is applied in 50 directions, b value is 0 scan 2 times, field of view (FOV): 240 × 240 mm, spatial resolution: 1 × 1 × 4 mm; echo time (TE):100 ms; repetition time (TR): 10 000 ms; layer thickness: 4 mm, layer spacing: 0 mm; flip angle (FA) 90°, scan time: 8′50″, scan layer number: 35 layers, a total of 1820 images were collected in the whole brain.

The studies involving human participants were reviewed and approved by the Zunyi Medical University Ethics Committee, Zunyi Medical University. Written informed consent to participate in this study was provided by the participants’ legal guardian/next of kin. Written informed consent was obtained from the individual(s), and minor(s)’ legal guardian/next of kin, for the publication of any potentially identifiable images or data included in this article.

### 2.3 Data preprocessing

All data have been preprocessed by a series of standard preprocessing procedures. dcm2niigui software was used to convert all the image formats from Dicom to 3D nifty. Then, the data differences between different sampling time points are compared and corrected according to the 6 displacement directions (Yaw, Pitch, Roll, DS, DL, and DP) so that the brain images of the same subject at each time point are unified to the same direction. The phase errors caused by eddy currents in the acquired images by eddy current correction were removed to reduce the effect of this error on the subsequent analysis. In order to achieve spatial normalization, the cranial and scalp parts of the non‐brain tissue were removed in the data preprocessing stage to eliminate interfering information. Finally, a child's brain image in our dataset is selected as a template, and SPM8 toolbox (statistical parametric mapping 8) in the MATLAB R2017a platform is utilized to register all the data.

### 2.4 Mask production

**FIGURE 1:**
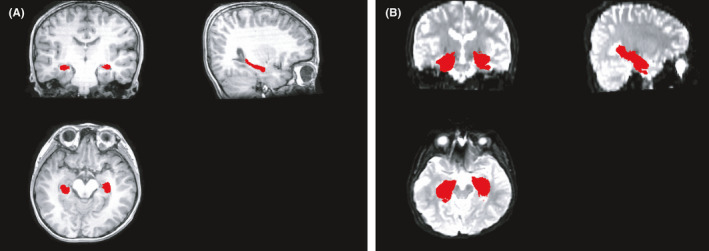
Produced mask of hippocampus. (A) The segmented hippocampus in T1 and (B) the produced mask of hippocampus

Then, we tried to make hippocampus mask. Segmenting the hippocampus region for each subject's T1 image was first performed, which were used to mask the hippocampus region of kurtosis tensor. Deep segmented CNN was employed to perform the segmentation, which was trained by Ataloglou et al.^27^ Then, since it contains T1 and segmented hippocampus images, the EADC‐ADNI HarP dataset (http://adni.loni.usc.edu/) was used to fine‐tune the network and segment the hippocampus region of T1 image for each subject. In order to fully cover the hippocampus area for all subjects, union operation was used to produce the hippocampus mask. Since it was found that there was little difference between the 3 age groups in the shape and size of the hippocampus, the hippocampus regions segmented by all subjects are used to calculate the hippocampus mask regardless of the age group. Figure 1 shows the produced mask of hippocampus.

### 2.5 Localization of epileptic foci with kurtosis tensor

#### 2.5.1 Framework

**FIGURE 2:**
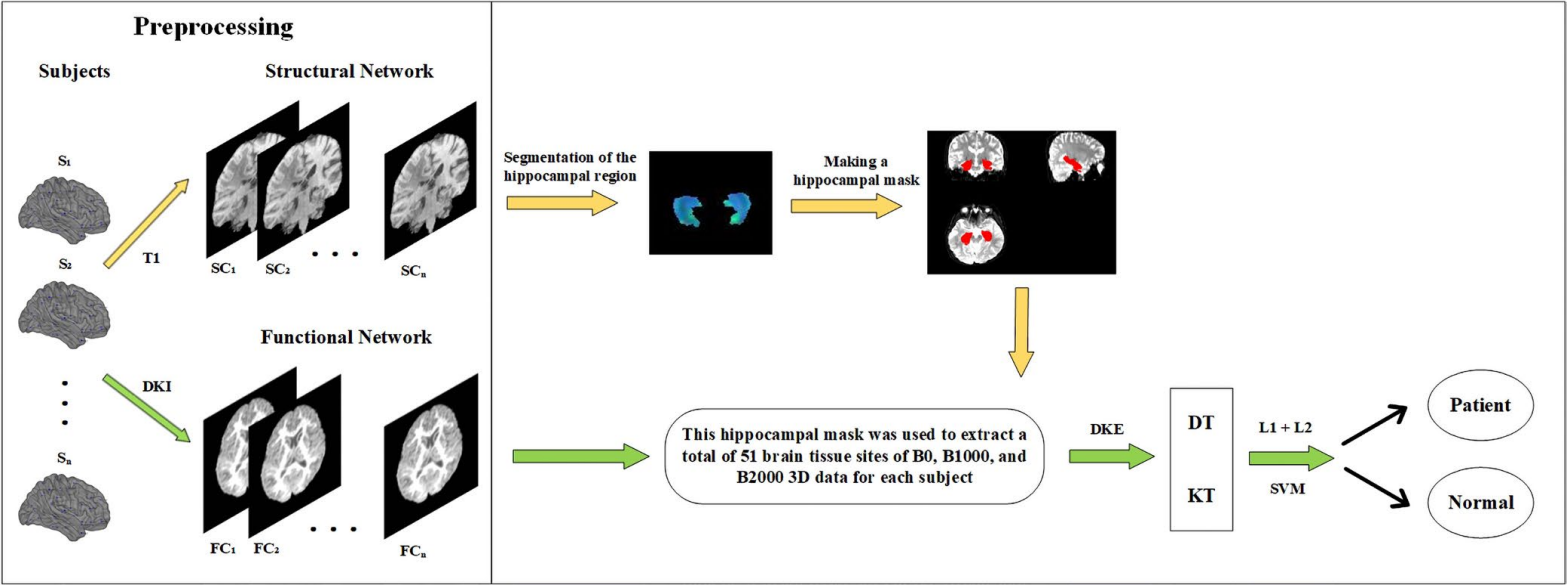
Flowchart for identifying epileptogenic foci

The flowchart of the proposed location method of epileptogenic foci is shown in Figure 2.

In the flowchart, the T1 and DKI images for each subject are preprocessed firstly. Then, we segment the hippocampal regions of the preprocessed T1 images of the brain and make the hippocampal masks based on the segmented hippocampal regions, which are subsequently utilized to extract the hippocampus for each subject's DKI images at B0, B1000, and B2000, and then, the kurtosis tensor of the hippocampus is estimated using DKE software. Since the feature extraction of the data is extremely critical to the performance of the algorithm, in order to balance data sparsity and algorithm stability, a combination of L1 and L2 regularization is finally used to normalize the model and extract effective features, which were fed into the SVM to identify epileptic patients from normal controls.

#### 2.5.2 The extraction of ROI and estimation of kurtosis tensor

In this article, we propose a new method to locate epilepsy by dividing brain tissue into several regions of interest, and then determining the presence or absence of lesions in each region of interest by classifying epileptic patients from normal controls. For the sake of simplicity and the data we collected, only the hippocampus was segmented as a local brain region in this article. Although only a single brain region was considered in this article, it should be noted that it is convenient to generalize the proposed approach to other brain regions and perform the localization of epileptic foci.

To extract the region of interest, we converted the data format of the T1 image from Dicom to 3D image with dcm2niigui software. Then, the affine transformation and interpolation of the 3D images of each subject under the parameters of B0, B1000, and B2000 were performed using the affine matrix, which is the transformation of each subject's image at B0 to its aligned T1 image. Subsequently, the kurtosis tensor of the extracted ROI is estimated.

To characterize anisotropic, non‐Gaussian diffusion dynamics, it is assumed in DKI that the diffusion‐weighted signal can be well described by the fourth‐order cumulant expansion of the diffusion signal, provided that the b value(the strength of diffusion weighting) is not too large. The natural logarithm of the diffusion signal is thus given by^28^:

(1) $$\ln S\left(b,\hat{n}\right)=\ln S_{0}‐b\sum_{\mathrm{ij}}n_{i}n_{j}D_{\mathrm{ij}}+\frac{b^{2}{\bar{D}}^{2}}{6}\sum_{\mathrm{ijkl}}n_{i}n_{j}n_{k}n_{l}W_{\mathrm{ijkl}}$$

where *b* is the *b* value, $\hat{n}$ is a normalized direction vector with the ‘hat’ symbol indicating a unit vector, $S_{0}$ is the signal with no diffusion weighting, *D* is the diffusion tensor, $\bar{D}$ is the mean diffusivity, *W* is the kurtosis tensor, the subscripts label Cartesian components, and sums on the indices are carried out from 1 to 3.

Directional diffusivity and diffusional kurtosis estimates for an arbitrary direction are given by:

(2) $$D\left(\hat{n}\right)=\pm \sum_{\mathrm{ij}}n_{i}n_{j}D_{\mathrm{ij}}$$

(3) $$K\left(\hat{n}\right)=\frac{{\bar{D}}^{2}}{D{\left(\hat{n}\right)}^{2}}\sum_{\mathrm{ijkl}}n_{i}n_{j}n_{k}n_{l}W_{\mathrm{ijkl}}$$

The diffusion tensor *D* is a 2‐dimensional tensor with size 3 × 3, and the diffusion kurtosis tensor *W* is a 4‐dimensional tensor with size 3 × 3 × 3 × 3. Due to the symmetry of the tensor, there are actually only six independent components in the diffusion tensor *D* and 15 independent tensors in the kurtosis tensor *W*. Consequently, the diffuse signal contains only 21 independent components.

#### 2.5.3 Feature extraction

In order to obtain the diffusion tensor (DT) and kurtosis tensor (KT) of the subjects, we segment the DKI images at B0, B1000, and B2000 to obtain the corresponding hippocampus for each subject, respectively. The segmented hippocampus for each subject contains 741 576 voxels; since DT for each voxel contains six independent components and KT contains 15 independent components, we shape the DT and KT of the hippocampus for each subject into the matrixes of order 6 × 741 576 and 15 × 741 576, respectively. Since the obtained matrix is highly sparse, we remove the all‐zero rows and all‐zero columns to obtain new matrices of order 6 × 16 000 and 15 × 16 000, respectively.

Although the aforementioned method reduces the amount of data significantly, however, the data remain sparse and most of the features are negligible in the classification task. To extract the most discriminative features that contribute to the classification task, a combination of L1 and L2 sparse regularization methods is developed in this article.

Suppose there are *N* samples and $X=(X_{1},X_{2},\ldots ,X_{N})$, $Y=(y_{1},y_{2},\ldots ,y_{N})$ are the labels of the *N* sample, feature selection can be represented as the following optimization model.

(4) $$\begin{array}{c} {\text{argmin}}_{\beta \in R^{P}}\frac{1}{N}{∥Y‐X\beta^{T}∥}_{2}^{2}\\ \;\;\;\;\;\;\;\;\;\;\;s.t.{∥\beta ∥}_{1}\leq t \end{array}$$

where $\beta =(\beta_{1},\beta_{2},\ldots ,\beta_{P})$ is the vector of regression coefficients under the sparse assumption. To constrain the model, we introduce regular terms that combine L1 and L2, which is shown as follows.

(5) $$\begin{array}{c} {\text{argmin}}_{\beta \in R^{P}}\frac{1}{N}{∥Y‐X\beta^{T}∥}_{2}^{2}+\lambda \rho {∥\beta ∥}_{1}+\lambda \frac{1‐\rho }{2}{∥\beta ∥}_{2}^{2}\\ \;\;\;\;\;\;\;\;\;\;\;\;\;\;\;\;\;\;\;s.t.\;\;{∥\beta ∥}_{1}\leq t\;\;\; \end{array}$$

where $\lambda_{\rho }{∥\beta ∥}_{1}$ is the penalty term of LASSO method, and λ is a non‐negative parameter, which is a linear model that estimates sparse coefficients. It is useful due to its tendency to prefer solution with fewer nonzero coefficients, effectively reducing the number of features upon which the given solution is dependent. At the same time, L2 norm, $\lambda \frac{1‐\rho }{2}{∥\beta ∥}_{2}^{2}$, is introduced to normalize the model in this article, which constrains the size of the model to avoid over‐fitting and make the model generalized to new classification task.

#### 2.5.4 Classification with SVM

Support vector machine (SVM) is the most popular classifier to deal with high‐dimensional small dataset, which seeks a maximum margin hyper‐plane to separate epileptic patients and normal controls. Given a training set ${\left(x_{k},y_{k}\right)}_{k=1}^{N}$ with input data $x_{k}\in R^{P}$ and corresponding binary class labels $y_{k}\in \left\{‐1,+1\right\}$, the output of primal SVM is presented as follows.

(6) $$y\left(x\right)=sign[w^{T}\varphi \left(x\right)+b]$$

where φ(x) is a non‐linear function to map the input data space to higher dimensional feature space, which could separate the input data linearly by the hyper‐plane. *b* is a bias term, and the optimization objective function can be defined as follows.

(7) $$\begin{array}{c} \text{min}\;J\left(w,\xi \right)=\frac{1}{2}w^{T}w+c\sum_{k=1}^{N}\xi_{k}\\ s.t.\;\;y_{k}\left[w^{T}\varphi \left(x_{k}\right)+b\right]\xi_{k},\;k=1,...,N,\;\xi_{k}\geq 0 \end{array}$$

$\xi_{k}$ is a slack variable, which indicates the tolerance of misclassification, *w* is the weight applied for input data *x*, and *c* is a tuning parameter and is a positive real constant.

## 3 EXPERIMENTAL RESULTS

To evaluate the classification performance of the proposed method in this article, the accuracy (ACC), precision (PRE), sensitivity (SEN), specificity (SPE), and area under the receiver operating characteristic curve (AUC) were used as metrics in the experiment, which are defined as follows.

(8) accuracy=(TP+TN)/(TP+TN+FP+FN)

(9) precision=TP/(TP+FP)

(10) sensitivity=TP/(TP+FN)

(11) specificity=TN/(TN+FP)

where TP and TN denote the number of positive cases predicted to be positive and negative cases predicted to be negative, respectively, and FP and FN denote the number of negative cases predicted to be positive and positive cases predicted to be negative, respectively.

We used the proposed method to identify the patients from normal controls and obtained the following results listed in Table 2.

**TABLE 2:** Results of classification patients and NC with the proposed method

	Train ACC	Test ACC	PRE	SEN	SPE	AUC
DT	0.9654	0.9375	0.9167	**0.9821**	0.8000	0.9600
KT	**0.9824**	**0.9524**	**0.9774**	**0.9821**	**0.9789**	**0.9900**

Boldface indicates the best results or important conclusion.

It can be seen from Table 2 that favorable results could be obtained whether DT or KT was used as the biomarker to classify epileptic patients and normal controls, which indicates that it is discriminative to identify epileptic patients from normal controls with DT or KT. In addition, the fact that KT is superior to DT on most of the indictors suggests that KT is more distinguishable than DT while applying to the recognition task on epilepsy.

## 4 DISCUSSION

### 4.1 Evaluation on the discrimination of tensor

Diffusion tensor and kurtosis tensor are two measures of microstructural changes in the brain. The study of epilepsy or other central nervous system diseases based on DTI or DKI generally utilizes parameters derived from DT or KT, such as FA, MD, or MK, while this article proposes to classify the epileptic patients from normal controls with the tensor directly. Consequently, we evaluated DT and KT for significant identification of epilepsy. Independent‐samples *t* test can be used to deduce the probability of the occurrence of differences, so as to compare whether the differences between two groups of data are significant. Specifically, firstly, we calculate the maximum, minimum, and average values of DT and KT tensors after feature extraction. Then, we collate and analyze all the above data for the independent‐samples *t* test; the results show that all data meet the requirement of normal distribution. Finally, independent‐samples *t* test was performed on the patient group and the normal control group, and the results are shown in Table 3.

**TABLE 3:** Results of independent‐samples *t* test results

	Normal (×10^−3^mm^2^/s)	Patient (×10^−3^mm^2^/s)	*p*
DT			
Max	3.8670 ± 0.7876	4.4452 ± 0.7972	**9.8e−10**
Min	−0.5096 ± 0.1718	−0.6767 ± 0.1244	**<2.2e−16**
Avg	0.5116 ± 0.1563	0.7025 ± 0.1048	**<2.2e−16**
KT			
Max	3.0766 ± 0.8837	2.4051 ± 0.6850	**1.3e−14**
Min	−0.9149 ± 0.2591	−0.7021 ± 0.5436	**7.5e−05**
Avg	0.2402 ± 0.0441	0.1743 ± 0.0089	**<2.2e−16**

Boldface indicates the best results or important conclusion.

It can be seen from Table 3 that the *p*‐values for DT and KT maxima, minima, and means were all ≤0.001, indicating that there were highly significant differences in DT and KT between patients and normal controls, and the differences in maxima, minima, and means were significant, indicating that DT and KT were better differentiated between the patients and normal controls.

**FIGURE 3:**
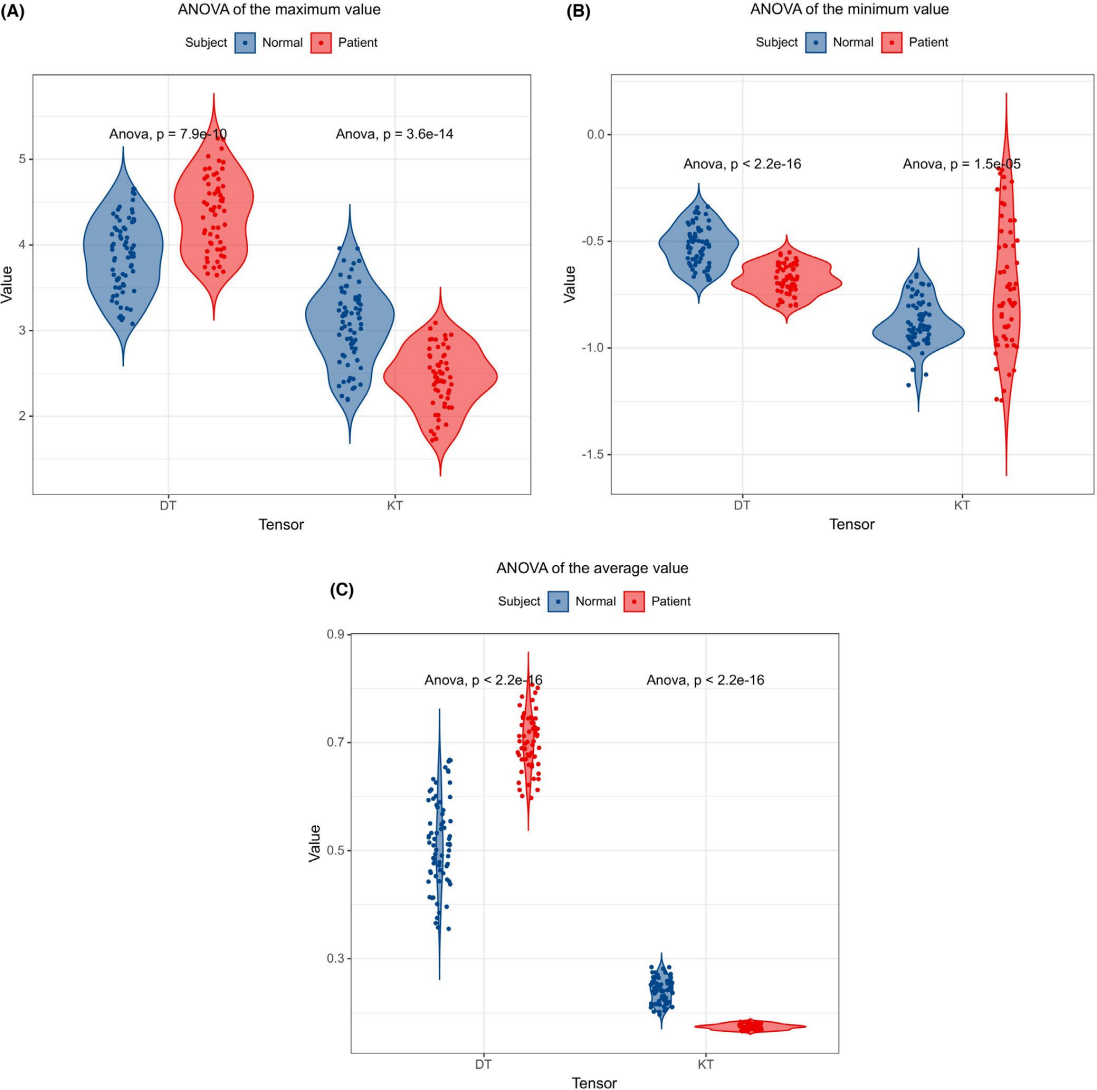
(A) Two‐violin diagram of the maxima values of DT and KT in patients and NC; (B) two‐violin diagram of the minimum values of DT and KT in patients and NC; (C) two‐violin diagram of the mean values of DT and KT in patients and NC

Additionally, ANOVA is used to test the significance of differences in the mean of two or more samples. To further indicate that there were significant differences in DT and KT between normal subjects and patients, ANOVA was performed on all subjects, and the maximum, minimum, and average values of DT and KT in patients and normal controls were obtained, respectively. The data results of ANOVA are shown in Figure 3, and the results show that all *p*‐values were ≤0.05, indicating that there were indeed significant differences in DT and KT between normal people and patients. In addition, Figure 3 shows a violin diagram of grouped data, where the scatter point represents the distribution of the maximum, minimum, and average values of DT and KT tensors after feature extraction for each subject.

### 4.2 Evaluation of feature extraction

Feature extraction technique is critical to the performance of classification, and we developed a feature extraction method combining L1 and L2 norms. In this section, we evaluate the effectiveness of the proposed method. In the experiment, firstly, LASSO and PCA were employed to extract features from DT and KT, respectively; then, the extracted features were fed into SVM to perform the classification task. Table 4 shows the results of classification.

**TABLE 4:** Evaluation on feature extraction

Method	DT	KT
×	0.9375	0.8750
L1	0.9375	0.9375
PCA	0.9375	0.9375
Ours	0.9375	**0.9524**

Boldface indicates the best results or important conclusion.

As can be seen from the results in Table 4, the accuracy of feature extraction with L1 penalty term, the combination of L1 and L2 penalty term, or PCA is 0.9375, which is the same as that without feature extraction. It is probably because that DT tensor has a low dimension, and dimension reduction has little effect on the improvement of accuracy. For KT, the accuracy of feature extraction using L1 penalty term and PCA was 0.9375, which was 6.25% higher than that without feature extraction. The method combining L1 and L2 penalty item achieves the highest accuracy of 95.24%, which is 7.74% higher than that without feature extraction, indicating that the combination of L1 and L2 penalty item has a better effect on KT feature extraction.

**FIGURE 4:**
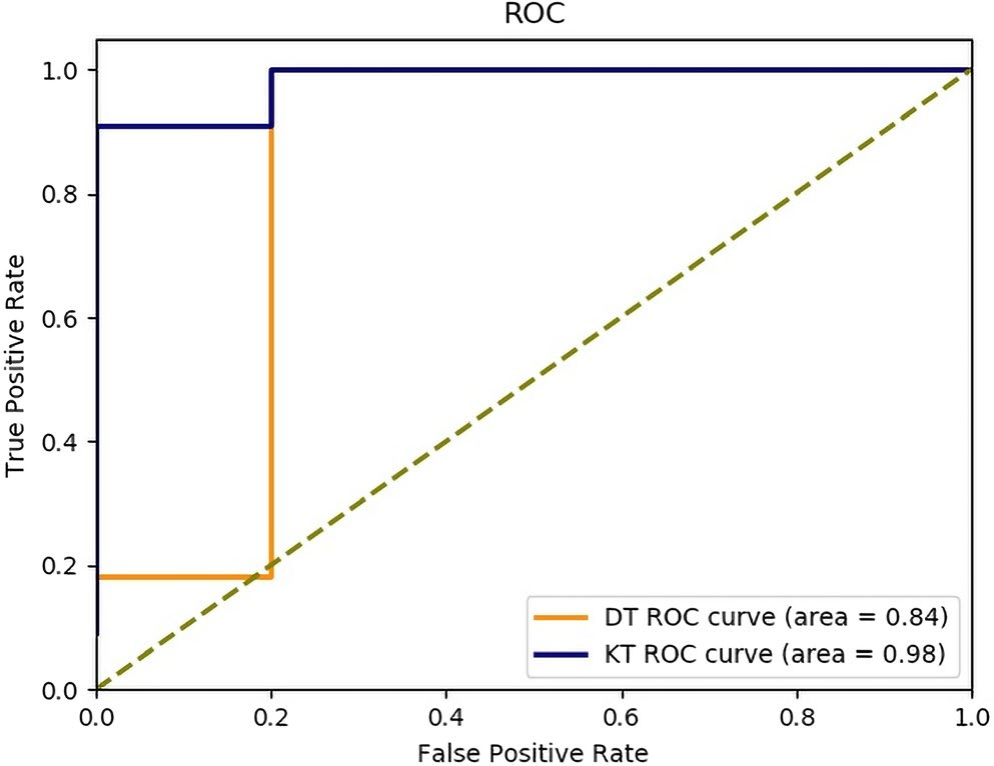
ROC of DT and KT

The ROC of DT and KT is depicted in Figure 4. We can see that the area under the ROC of DT was 0.84, while the area under ROC of KT could reach 0.98, indicating that the diagnostic effect of KT was much better than that of DT, and further proving the superiority of DKI kurtosis tensor for the localization of conventional MRI‐negative epileptic focus. It should be noted that it is normal for the ROC curve in this section to take the shape of step, because the experiment in this section is implemented on individuals, and the data set is relatively small, which leads to the phenomenon of unsmoothness.

### 4.3 Comparison with other studies

Most of studies previously use parameters, such as FA, MK and MD, derived from DT or KT to analyze the central nervous system disease, while this article proposes a new approach to perform the data analysis with KT, which is different from the conventional methods. Consequently, we test the performance of FA, MK, MD, DT, and KT on the recognition of epilepsy, and the results are shown in Table 5.

**TABLE 5:** Comparison between parameters and tensor for distinguishing patients from NC

Item	Train ACC	Test ACC	PRE	SEN	SPE	AUC
FA	0.9519	0.8873	0.8899	0.8661	0.9059	0.9600
MD	0.8166	0.5887	0.5763	0.4554	0.7059	0.6200
MK	0.9679	0.9392	0.9257	0.9242	0.9436	0.9800
FA + MK	0.9727	0.9268	0.9116	0.9235	0.9295	0.9700
DT	0.9654	0.9375	0.9167	**0.9821**	0.8000	0.9600
KT	**0.9824**	**0.9524**	**0.9774**	**0.9821**	**0.9789**	**0.9900**

Boldface indicates the best results or important conclusion.

**FIGURE 5:**
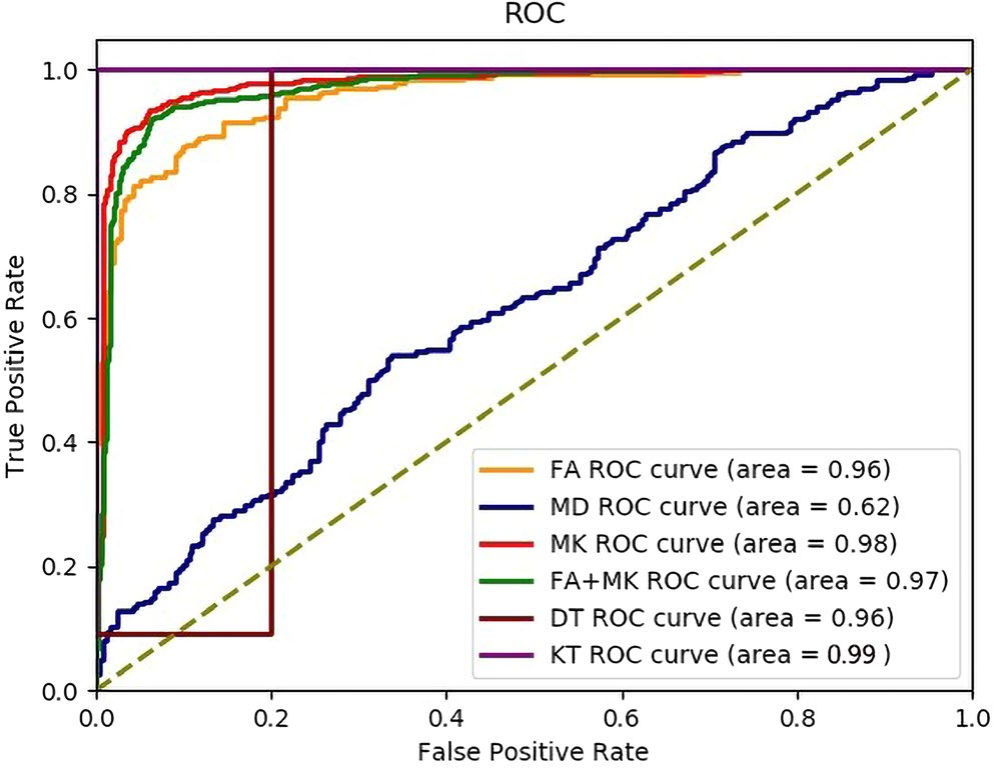
ROC of the proposed method and other method based on parameters

It can be seen from Table 5 that the accuracy of classification with KT in the test set was higher than the highest value of 0.9392 with parameter MK, and the other indicators, precision, sensitivity, specificity, and AUC with KT were also outstanding. The accuracy with DT reached 0.9375, slightly lower than that with parameter MK, but sensitivity reached 98.21%. As we all know, sensitivity is much more important than specificity when making a diagnosis. It is prefer to diagnose a healthy person as a patient than to diagnose a patient as a healthy one, which may delay the treatment and lead to the deterioration of the condition. We also depicted the ROC of the proposed method and other method based on DKI, as shown in Figure 5.

On the whole, distinguishing epileptic patients from NC with DT or KT is superior to those methods with the parameters derived from DT and KT, which demonstrates it is feasible to analyze the brain microstructural changes of epilepsy with DT or KT directly, instead of calculating the parameters from these tensors.

In addition, the classification performance of the proposed method in this study was compared with studies based on other modalities, and the results are shown in Table 6.

**TABLE 6:** Classification accuracy of the proposed method compared with other studies

Method	Modality	ACC
Bhattacharyya et al.^29^	EEG	82.53%
Amarreh et al.^30^	DTI	83.90%
Zhang et al.^31^	fMRI	85.00%
Acharya et al.^32^	EEG	88.67%
Chatterjee et al.^33^	EEG	92.18%
Sharma et al.^34^	EEG	95.00%
**Ours**	**DKI**	**95.24%**

Boldface indicates the best results or important conclusion.

The results listed in Table 6 were obtained with single modality approaches, such an EEG, DTI and fMRI, and CNN or SVM was employed to classify the epileptic patients and NC. Regardless of the modality and techniques employed in these methods, the proposed method achieves better classification accuracy compared with the other modality‐based recognition algorithms.

## 5 CONCLUSION

Epilepsy is a serious hazard to human health, and it is critical to identify the epileptic foci for the subsequent treatment. For the recognition of epileptic foci of MRI‐negative patients, this article presents a method based on diffusion kurtosis tensor, which could identify epileptic patients from normal controls in a single brain region, especially the hippocampus as an example. Although only a brain region is considered in this article, it should be noted that this method could be generalized to other suspected brain regions and then locate the lesion accordingly.

Most of other studies based on DKI employ the parameters, such as FA, MK, and MD, derived from the diffusion tensor or kurtosis tensor as the biomarker to analyze epilepsy; however, as we all know, diffusion tensor and kurtosis tensor themselves should contain more complete information about the microstructure of the brain. Accordingly, we propose to identify epileptic patients from normal controls with the kurtosis tensor directly instead of these derived parameters. In addition, machine‐learning technique is introduced to perform the classification task in this article and to extract features more exactly, especially the combination of L1 and L2 regularization is developed to normalize the model. Several experiments were set up to verify the effectiveness of classifying epileptic patients from normal controls with kurtosis tensor and the proposed learning method (Supplementary Material).^29^, ^30^, ^31^, ^32^, ^33^, ^34^ From the results, it can be seen that the performance of classification with kurtosis tensor is indeed superior to other methods, and even if compared with other imaging modality, the proposed method also produced favorable results. All these illustrate that both diffusion kurtosis tensor and the classification method based on machine learning are promising in the identification of epileptic foci.

Although the method proposed in this article yields better results in classifying epilepsy and normal controls, it should be noted that, in contrast to the state‐of‐the‐art methods, it allows indirect localization of epileptogenic foci, since we can locate epileptic foci by analyzing other suspected brain regions with the same method and then locate the lesion according to the classification results. As an example, if we are not sure where the lesion is, we can input the patient's kurtosis tensor into the segmentation convolutional neural network or other segmentation software, divide the kurtosis tensor into multiple brain regions, and then input the images of each brain region into the feature extraction and classification module to make predictions and judgments; the location of the lesion may provide imaging reference for the study of the pathophysiological mechanism of epilepsy, and subsequently, the epileptic foci could be located based on the proposed method.

Overall, the most crucial contribution of this article is to verify that the kurtosis tensor in DKI is more discriminative for epilepsy recognition than FA, MK, and MD parameters, which will be a promising conclusion in the computer‐aided diagnosis of epilepsy based on diffusion imaging.

## CONFLICT OF INTEREST

We declare that we have no financial and personal relationships with other people or organizations that can inappropriately influence our work, and there is no professional or other personal interest of any nature or kind in any product, service, and/or company that could be construed as influencing the position presented in, or the review of the manuscript.

## Supporting information

Supplementary Material

## Data Availability

Some or all data, models, or code generated or used during the study are proprietary or confidential in nature and may only be provided with restrictions.
